# Developing a rapid-response program for health system decision-makers in Canada: findings from an issue brief and stakeholder dialogue

**DOI:** 10.1186/s13643-015-0009-3

**Published:** 2015-03-11

**Authors:** Michael G Wilson, John N Lavis, Francois-Pierre Gauvin

**Affiliations:** McMaster Health Forum, McMaster University, Hamilton, Canada; Centre for Health Economics and Policy Analysis, McMaster University, Hamilton, Canada; Department of Clinical Epidemiology and Biostatistics, McMaster University, Hamilton, Canada; Department of Political Science, McMaster University, Hamilton, Canada; Department of Global Health and Population, Harvard School of Public Health, Cambridge, USA

**Keywords:** Knowledge translation, Health systems, Rapid synthesis, Policymakers, Stakeholder dialogue

## Abstract

**Background:**

There is currently no mechanism in place outside of government to provide rapid syntheses of the best available research evidence about problems, options and/or implementation considerations related to a specific health system challenge that Canadian health system decision-makers need to address in a timely manner. A ‘rapid-response’ program could address this gap by providing access to optimally packaged, relevant and high-quality research evidence over short periods of time (i.e. days or weeks).

**Methods:**

We prepared an issue brief that describes the best available research evidence related to the problem, three broad features of a program that addresses the problem and implementation considerations. We identified systematic reviews by searching for organization-targeted implementation strategies in Health Systems Evidence (www.healthsystemsevidence.org) and drew on an existing analytical framework for how knowledge-brokering organizations can organize themselves to operationalize the program features. The issue brief was then used to inform a half-day stakeholder dialogue about whether and how to develop a rapid-response program for health system decision-makers in Canada. We thematically synthesized the deliberations.

**Results:**

We found very few relevant systematic reviews but used frameworks and examples from existing programs to 1) outline key considerations for organizing a rapid-response program,, 2) determine what can be done in timelines ranging from 3 to 10 and 30 business days, and 3) define success and measure it. The 11 dialogue participants from across Canada largely agreed with the content presented in the brief, but noted two key challenges to consider: securing stable, long-term funding and finding a way to effectively and equitably manage the expected demand. Recommendations and suggestions for next steps from dialogue participants included taking an ‘organic’ approach to developing a pan-Canadian network and including jurisdictional scans as a type of product to deliver through the program (rather than only syntheses of research evidence).

**Conclusions:**

Dialogue participants clearly signalled that there is an appetite for a rapid-response program for health system decision-makers in Canada. To ‘organically’ build such a program, we are currently engaging in efforts to build partnerships and secure funding to support the creation of a pan-Canadian network for conducting rapid syntheses for health system decision-makers in Canada.

**Electronic supplementary material:**

The online version of this article (doi:10.1186/s13643-015-0009-3) contains supplementary material, which is available to authorized users.

## Background

A gap exists in efforts to support the use of research evidence between ‘self-serve’ approaches (e.g. ‘one-stop shops’ for research evidence such as Health Systems Evidence - www.healthsystemsevidence.org) and ‘full-serve’ approaches (e.g. convening stakeholder dialogues with health system leaders who are informed by an evidence brief that synthesizes the best available research evidence). A ‘rapid-response’ program could fill this gap in situations where health system decision-makers need support with accessing and synthesizing optimally packaged, relevant and high-quality research evidence over days or weeks (i.e. when the timeline is too short to prepare a full evidence brief and convene a stakeholder dialogue).

When looking for support to find and synthesize research evidence in a timely manner, decision-makers may turn to internal research-support services (many of which use less systematic and transparent processes than are typically used by formally designated programs) or to researchers with whom they have an established relationship. Alternatively, they may turn to one of very few formalized rapid-response programs in Canada, if their question is about drugs and other health technologies [1], if they are in Quebec and have a question about drugs and other health technologies or about health and social programs and services [2], if they are in Ontario and pose a question related to HIV programs and services [3] or if they are a manager or stakeholder working within the Champlain Local Health Integration Network [4,5]. However, there is currently no mechanism in place outside of government to provide rapid syntheses of the best available research evidence about problems, options and/or implementation considerations related to a specific health system challenge (as opposed to a challenge with a program, service or drug).

Depending on the timelines provided, products provided through a rapid-response program might include a listing of relevant research evidence (if the timeline is very short), a brief synthesis of the results (if the timeline permits) or a more detailed summary (if given a longer period of time). Some rapid-response programs also supplement these products by conducting briefings with decision-makers based on the research evidence identified [6]. One added benefit of providing rapid-response programs is that decision-makers who have previously used such services and found them to be valuable may be more inclined to think about finding and using research evidence in the future, and/or highlight the value of doing so to their peers. Another added benefit is that the products of a rapid-response program (which we will call ‘rapid syntheses’) can be made available in a repository for others to access (as effort to facilitate ‘user pull’ for research evidence) or be actively disseminated to policymakers in other settings (as a ‘push’ mechanism) who may (or eventually will) be grappling with the same or similar issues.

We see rapid syntheses as being distinct from a rapid systematic review (and other variants such as rapid realist review) in several ways. First, those requesting a rapid synthesis typically set the timeline within which it needs to be prepared (typically no more than a few weeks). In contrast, a rapid review is typically a comprehensive systematic review conducted in a condensed timeline (e.g. 6 months), rather than a more standard timeline like 1 or 2 years. Second, the nature of the questions addressed can take many forms, and relate to a problem, options or implementation considerations, as opposed to a rapid review of the effects of a single option. Lastly, rapid syntheses typically include existing systematic reviews and occasionally single studies, whereas rapid reviews focus on single studies.

We recently created a rapid-response program at the McMaster Health Forum that provides a summary (what we call a ‘rapid synthesis’) of research evidence based on a systematic search for information about problems, options and/or implementation considerations related to a specific health system challenge [7]. At present, current funding allows us to complete four requests (over a 3, 10 or 30 business day time frame) per year for Ontario-based knowledge users (e.g. policymakers, managers of healthcare institutions, community-based organizations) who are involved in decision-making about health systems. Given that this service would likely benefit knowledge users across the country, we convened a half-day stakeholder dialogue in March 2014 focused on whether and how to develop a rapid-response program for health system decision-makers in Canada. As outlined in the discussion, based on feedback from dialogue participants, requests can now be taken from knowledge users outside of Ontario on a cost recovery basis and additional funding is being sought to allow us to complete a greater number of requests each year.

## Methods

Convening stakeholder dialogues is one promising approach to addressing health system issues (in this case, how to best support timely access to the best available research evidence about pressing health system issues). Stakeholder dialogues are designed to support evidence-informed decisions by pairing the best available research evidence with a robust deliberative process that gives stakeholders the opportunity to bring their tacit knowledge and their own views and experiences to bear on a pressing health system problem, three options to address it and implementation considerations [8]. Generally, convening health system stakeholders (e.g. government officials, professional and community leaders, patients/citizens and groups representing them, and researchers) for deliberations has the overarching goal of supporting participants to champion creative efforts to address a pressing health system problem within their respective constituencies [8]. Each dialogue is informed by an evidence or issue brief that mobilizes the best available research evidence about the problem, options and implementation considerations. While using the same approach as an evidence brief, issue briefs, such as the one produced for this dialogue, draw on findings from a previously conducted synthesis of the evidence or in the absence of syntheses uses analytical frameworks to help dialogue participants critically engage with the topic [8].

We describe below our methods for preparing the issue brief and convening the dialogue. For those who are interested in more detail, a separate paper outlines the overall approach for developing evidence/issue briefs and convening stakeholder dialogues [8].

### Preparing the issue brief

We prepared the issue brief through four steps: 1) convening a steering committee; 2) developing and refining a terms of reference for an issue brief; 3) identifying, selecting, appraising and extracting key findings from the research evidence about the problem, program features and implementation considerations; and 4) synthesizing the findings in the form of an issue brief. Our steering committee was comprised of representatives from partner organizations and stakeholder groups and provided guidance throughout the process as well as literature that would be relevant to preparing the brief. The terms of reference for the issue brief provided a preliminary outline to clarify the problem, framed three options for addressing it (in this case, three broad features of a program for addressing the problem given the nature of the topic being addressed) and identified implementation considerations. We developed and iteratively refined the terms of reference in collaboration with our steering committee.

For the third stage, we identified relevant systematic reviews related to the three program features (organizing a rapid-response program, establishing what can be done in what timelines and defining success and measuring it) by searching Health Systems Evidence (www.healthsystemsevidence.org), which is a continuously updated database, that contains (as of January 2015) 4,200 systematic reviews and more than 2,200 economic evaluations of delivery, financial and governance arrangements within health systems. For more information about the sources searched to identify documents included in Health Systems Evidence, see http://www.healthsystemsevidence.org/why-use-it.aspx. The reviews and economic evaluations were identified by searching the category for organization-targeted implementation strategies in Health Systems Evidence. The searches were reviewed by one of us (MGW) with another (JNL) checking reviews for which there was uncertainty about their inclusion. For each systematic review, we extracted the focus of the review, key findings, the last year the literature was searched, the methodological quality (based on AMSTAR [9] ratings that are provided for all reviews contained in Health Systems Evidence), the proportion of included studies that were conducted in Canada and the proportion of included studies focused explicitly on supporting the use of research evidence. For any reviews that had not been previously quality appraised using AMSTAR, two reviewers (MGW and FPG) independently completed an assessment.

Given the nature of the topic, there are very few relevant systematic reviews available, so we drew heavily on an existing analytical framework for how knowledge-brokering organizations can organize themselves [10]. We then used this framework to derive possible organizational features of a rapid-response program for health system decision-makers in Canada and to document features of other rapid-response programs that are focused on addressing questions related to health systems (see the Results section for more about the framework and our application of it). These other programs were identified through our own knowledge of existing programs and by asking our steering committee and key contacts about programs they were aware of. We then conducted hand searches of each program website to document organizational characteristics related to the key domains of the analytical framework we noted above (these broadly related to governance, management and staffing, program resources and collaborations) as well as key features of their methods and products.

Lastly, we drafted the brief by presenting the evidence we identified in concise and accessible language. The final version of the briefs consisted of a one-page summary of key messages followed by a more detailed description of 1) the problem, 2) three broad features of a program that could address the problem and 3) possible barriers to implementation of the options at the levels of individuals, providers, organizations and systems. The brief was then merit reviewed by a small number of policymakers, stakeholders and researchers to ensure its system relevance and scientific rigour.

### Convening the stakeholder dialogue

We worked collaboratively with the steering committee to identify health system stakeholders (for this dialogue, principally, government officials as well as some stakeholders who are or have been involved in developing and administering a rapid-response program). For each dialogue we convene, we invite those who have the ability to 1) bring unique views and experiences to bear on the challenge and learn from the research evidence and from others’ views and experiences and 2) champion within their respective constituencies the actions that will address the challenge creatively. Participants were principally identified from suggestions provided by members of the steering committee, which we supplemented by reviewing government directories and websites of relevant organizations.

The dialogue was facilitated by one of us (JNL) and included deliberations about the same topics addressed in each of the three sections of the brief (problem, program features and implementation considerations). These were followed by a fourth deliberation about the next steps that could be taken. Participants were sent the brief 2 weeks before the dialogue and were requested to read it before arriving. The goal was not to aim for consensus *per se*, but rather provide a space where diverging opinions could be shared and discussed and to identify possible synergistic efforts among stakeholders. In addition, the dialogue followed the Chatham House Rule (i.e. ‘the information received during the meeting can be used, but neither the identity nor the affiliation of the speaker(s), nor that of any other participant, may be revealed’) [11]. Lastly, each of us took notes during the deliberations as we did not audio record the dialogue. We used these notes to draft a summary of the dialogue by highlighting the key themes that emerged during each deliberation, points of disagreement or general consensus and the types of action that participants thought could be taken following the dialogue. We adhered to the Chatham House Rule by keeping the identity of participants confidential in writing the summary.

## Results

We present below a summary of the key findings from the issue brief and the key themes of the deliberations. For those who are interested in more information about each, the issue brief [12] and dialogue summary [13] are freely available on the McMaster Health Forum website (www.mcmasterhealthforum.org).

### Key findings from the issue brief

#### The problem

Despite a range of approaches that are available in Canada to support health system decision-makers’ efforts to find and use research evidence efficiently, significant barriers and challenges exist. We identified the following as key components of the problem:

- limited number of formalized supports in place to provide decision-makers with rapid syntheses of the best available research evidence about problems, options and/or implementation considerations related to health system challenges (i.e. providing the right product at the right time);
- inconsistent interaction between researchers and decision-makers to ensure that the priorities of decision-makers are addressed (i.e. having the right people developing products on the right issues); and
- uncertainty about what success looks like given the long chain of potential causal relationships between an intervention/program (e.g. a rapid-response program) and relevant outcomes (e.g. whether decision-makers’ needs are met and/or their use of research evidence).

#### Three broad features of a program to address the problem

To promote discussion during the stakeholder dialogue about the pros and cons of a potentially viable approach to developing a rapid-response program for health system decision-makers in Canada, we identified three broad program features. The three program features were developed and refined through consultation with the steering committee and include activities related to 1) organizing a rapid-response program, 2) establishing what can be done in what timelines and 3) defining success and measuring it.

We did not identify any systematic reviews related to organizing a program to support the use of research evidence (program feature 1). What we do know is that it is essential to match form to function when organizing such a program [10]. To do so (and to foster deliberations about doing so), we identified four organizational features (governance, management and staffing, resources and collaboration) from a recent policy summary designed to encourage debate and innovation about the ways in which knowledge-brokering organizations organize themselves [10]. According to this policy summary, knowledge brokering refers to the ‘use of information-packaging mechanisms and/or interactive knowledge-sharing mechanisms to bridge policy-makers’ and researchers’ contexts’, which encompasses many of the proposed activities of the rapid-response program. We outline these types of organizational features in Table 1 along with possible approaches to operationalizing each of them.

**Table 1:** **Summary of organizational features and possible approaches to operationalizing them (table from Wilson et al. 2014)** [12]

**Organizational feature**	**Possible approaches to operationalizing each feature**	**Criteria met** ^a^
Governance (structure, scope and rules)	● Administer the rapid-response program through the McMaster Health Forum under its existing governance structure that prioritizes strong links with and involvement of policymakers and stakeholders in the programs it delivers	● 1, 7
	● Operationalize this approach to governance by convening a rapid-response program steering committee consisting of federal, provincial and territorial health system decision-makers and stakeholders who can provide strategic guidance about administering the program	● 1, 9
	● Establish that the rapid-response program:	● 2
	○ addresses topics requested by health system decision-makers (requests will be submitted to the Forum through email and the questions will be refined by the Forum in collaboration with the requestor where necessary);	
	○ ensures that the findings of the syntheses are based on the available research evidence and not the personal views of those who requested or developed it;	
	○ identifies whether any potential conflicts of interest exist in any product produced through the rapid-response program; and	
	○ disseminates completed syntheses (e.g. through the existing Forum Update newsletter and/or through a dedicated email list to the program partners) and makes them available through a dedicated repository on the Forum’s website (but without the requestor’s jurisdiction attached to the synthesis to provide some level of anonymity)	
Management and staffing	● Allocate authority to the organizational leadership of the Forum for ensuring the accountability of the program in relation to its mandate	● 3
	● Use effective project management processes to make the best use of available resources and to sequence and prioritize tasks in a way that allows for all requests to be completed within specified timelines	● 6
	● Implement minimum training standards (e.g. completing an online training course about finding and using research evidence) and provide ongoing mentorship for staff contributing to the rapid-response program (this includes both those at the Forum and from partner networks or organizations)	● 4
Program resources	● Seek external (but not user-pay) and long-term funding (e.g. from a Partnerships for Health System Improvement grant from the Canadian Institutes of Health Research) that will allow for both the delivery and ongoing evaluation of the program	● 5 (if successful)
	● Prioritize some requests over others in times when demand exceeds available resources, which could be accomplished through one or more of the following approaches:	● 6
	○ completing requests from those who have not recently accessed the program;	
	○ requesting a resubmission at a later date for topics that are deemed less urgent (either by the requestors themselves, by the steering committee or both); and/or	
	○ engaging the program steering committee to help decide which requests should be prioritized (e.g. through a voting or ranking process over email)	
Collaboration	● Engage trusted national, provincial and territorial partner networks or organizations (where possible and necessary) to	● 8
	○ identify whether a synthesis has already been completed on the topic (e.g. by establishing a listserv that can be used to efficiently contact all partners when a request is received) and	
	○ collaborate with the Forum to conduct syntheses (or build on existing products identified) to ensure relevance to particular provincial and territorial contexts	

^a^The ordering of bullets in this column corresponds to the order in the adjacent column that lists possible approaches to operationalizing each feature.

The same policy summary also provides a set of nine criteria for assessing organizational models for knowledge brokering, which were derived from a multi-method study consisting of a systematic review, scoping review of knowledge-brokering mechanisms and models, website review of existing knowledge-brokering organizations, sites visits and case studies. The summary was designed for those involved in establishing or leading organizations that support the use of research evidence in developing health policy [10]. The following nine criteria have been extracted directly from Lavis et al. 2013 and ask whether a knowledge-brokering organization [10]

1. gives policymakers, stakeholders and researchers an explicit role in its governance and ensures they exercise their role with transparency and objectivity;
2. has and enforces rules that ensure independence in how health system information is produced, packaged and shared and that address conflicts of interest;
3. grants the director the authority needed to ensure the accountability of the entire organization to its knowledge-brokering mandate;
4. ensures an appropriate size, mix and capacity of staff with knowledge-brokering responsibilities;
5. ensures an appropriate size of budget and an appropriate mix of funding sources for knowledge-brokering activities;
6. has an explicit approach to prioritizing knowledge-brokering activities and accepting commissions or requests from policymakers and stakeholders;
7. is located within another organization or network that supports its knowledge-brokering activities;
8. collaborates with other knowledge-brokering organizations in its knowledge-brokering activities; and
9. establishes functional linkages with policymaking and stakeholder organizations.

In Table 1, we outline which of these criteria are met by the possible approaches to operationalizing each program feature.

We also documented features of several existing rapid-response programs that target, at least in part, health system decision-makers. We identified the programs in collaboration with our steering committee as well as from reviews of mechanisms designed to promote the use of research evidence by policymakers [14,15] and from our first-hand knowledge of existing programs. We only included formally organized programs that are designed to conduct rapid syntheses as their core task (as opposed to support that may be offered informally within government ministries or other organizations) given that we were interested in how to formally operationalize a rapid-response program. We documented the organizational features by reviewing their respective websites, through our first-hand knowledge of some of them, or based on input received from our project steering committee. In the cases where we relied on website review, informational gaps in our analysis may exist. We provide a list of the programs and their organization features in Additional file 1.

For program feature 2 (deciding what can be done in what timelines), we identified three different timelines in which a request can be made to the rapid-response program (3, 10 or 30 business days), which we summarize in Table 2. We identified several systematic reviews evaluating interventions for supporting the use of research evidence by policymakers, but each found insufficient evidence to draw conclusions about the effectiveness of interventions [16-19]. While the evidence is limited, one recent low-quality review found evidence to suggest that tailored targeted messages combined with access to registries of research evidence may increase the use of research evidence in policymaking [17]. To supplement the limited synthesized research evidence, we built on the profile of organizational characteristics of rapid-response programs provided in Additional file 1 by summarizing in Additional file 2 their target audience, types of topics addressed and the products provided (and the timelines in which they are produced).

**Table 2:** **Summary of what can and cannot be done in what timelines (table from Wilson et al. 2014)** [12]

**Timeline**	**What can be done**	**What cannot be done**
Three business days	● Identify systematic reviews and economic evaluations relevant to health systems from key databases (e.g. Health Systems Evidence)	● Identify primary research studies (e.g. published studies and unpublished reports)
	● Provide summary tables that outline	● Conduct quality appraisals for reviews that are not available through Health Systems Evidence
	○ key findings from relevant systematic reviews,	
	○ quality appraisals of systematic reviews (for reviews that are available through Health Systems Evidence) and	● Prepare a detailed summary of key findings
	○ countries in which studies included in systematic reviews were conducted (for reviews that are available in Health Systems Evidence)	● Engage experts to conduct a merit review of the findings to ensure scientific rigour and system relevance
		● Conduct jurisdictional scans of what is being done nationally and internationally
		● Conduct a full systematic review
Ten business days	● Identify systematic reviews and economic evaluations relevant to health systems from key databases (e.g. Health Systems Evidence)	● Identify grey literature (e.g. unpublished reports) that is not already contained in key databases (e.g. Health Systems Evidence)
	● Identify relevant primary research studies when limited evidence is available from systematic reviews	● Prepare a detailed summary of key findings
	● Provide summary tables that outline	● Incorporate feedback from experts engaged in the merit-review process within the 10-day timeline (but a final summary that incorporates reviewers’ feedback will be sent within another five business days)
	○ key findings from relevant systematic reviews,	
	○ quality appraisals of systematic reviews (for reviews that are available through Health Systems Evidence) and	
	○ countries in which studies included in systematic reviews were conducted (for reviews that are available in Health Systems Evidence)	
	● Prepare a brief summary of the key findings from systematic reviews (and primary research studies where relevant)	● Conduct jurisdictional scans of what is being done nationally and internationally
	● Engage experts to conduct a merit review of the brief summary to ensure scientific rigour and system relevance (a draft summary will be submitted to the requester before merit reviewer feedback is received and then a final summary that incorporates reviewers’ feedback will be submitted within another five business days)	● Conduct a full systematic review
30 business days	● Identify systematic reviews and economic evaluations relevant to health systems from key databases (e.g. Health Systems Evidence)	● Conduct a full systematic review
	● Identify relevant primary research studies when limited evidence is available from systematic reviews	
	● Conduct jurisdictional scans of what is being done nationally and internationally through targeted searches of databases for published literature, and websites of relevant jurisdictions and stakeholders for grey literature that is not already contained in key databases (e.g. Health Systems Evidence)	
	● Consult with experts with knowledge of the topic to identify additional relevant research evidence (contingent on locating relevant experts)	
	● Provide summary tables that outline	
	○ key findings from relevant systematic reviews	
	○ quality appraisals of systematic reviews (for reviews that are available through Health Systems Evidence) and	
	○ countries in which studies included in systematic reviews were conducted (for reviews that are available in Health Systems Evidence)	
	● Prepare a detailed summary of the key findings from systematic reviews (and primary research studies where relevant)	
	● Engage experts to conduct a merit review of the detailed summary to ensure scientific rigour and system relevance and incorporate reviewers’ feedback in the final report within the 30-business-day timeline	

For program feature 3 (defining success and measuring it), we identified four short- and medium-term areas where the success of a rapid-response program can be measured using a brief survey administered following receipt of a rapid synthesis and short interviews approximately 6 months later. The four areas of success include 1) program organization (i.e. whether the program is organized in a way that allows health system decision-makers to efficiently make a request and receive a timely response), 2) final product (e.g. was the synthesis presented in way that was easy to understand?), 3) influence on behavioural intention to use research evidence, and 4) whether and how the synthesis was used. In Table 3, we outline each of these potential areas of success and pair them with approaches to measuring whether we have been successful.

**Table 3:** **Summary of possible indicators of success and approaches to measuring success (table from Wilson et al. 2014)** [12]

**Where to measure success**	**Possible approaches to measuring whether we have been successful**
Program organization	● Brief survey asking the requestor to evaluate key features of the rapid-response program (administered after receipt of rapid synthesis)
	● Short qualitative interviews with requestors (conducted approximately 6 months following receipt of rapid synthesis)
Final product (i.e. did the rapid synthesis meet the requestor’s needs?)	● Brief survey asking the requestor to evaluate key features of the rapid synthesis
	● Short qualitative interviews with requestors asking questions about what was most and least helpful about the synthesis (6 months following receipt of rapid synthesis)
Influence on behavioural intention to find and use research evidence	● Assessment of behavioural intention (and the attitudes, social norms and perceived behavioural control that influence whether such intention translates into action) after receiving the rapid synthesis and 6 months later (assessed in survey administered after receipt of rapid synthesis and again during the short qualitative interviews 6 months later)
Whether and how the synthesis was used (i.e. did it support evidence-informed decision-making?)	● Short qualitative interviews with requestors about how they used the rapid synthesis (conducted 6 months following receipt of rapid synthesis)

The only systematic reviews we identified for this program feature related to the use of the theory of planned behaviour. One older low-quality systematic review and an older overview of systematic reviews from the psychology field found that the theory explains approximately 39% of the variance in intention and about 27% of the variance in behaviour [20,21]. Linkages of a similar magnitude between intention and behaviour among healthcare professionals were found in another older but high-quality systematic review [22], which lends support to it being used in the study of health system decision-makers [23].

#### Implementation considerations

We identified several possible barriers to implementing the program features, which we list in Table 4. In addition, we identified possible windows of opportunity for each program feature, which include

**Table 4:** **Potential barriers to implementing program features (table adapted from Wilson et al. 2014)** [12]

**Levels**	**Potential barriers**
Individual	● No barriers identified at the citizen or patient level for any of the program features
Service provider	*Program feature 1 - organizing a rapid-response program*
	● Existing providers of rapid-response programs may overlap to some extent with the scope of a new program focused on producing rapid syntheses for health system decision-makers about problems, options and/or implementation considerations related to a specific health system challenge
	*Program feature 2 - deciding what can be done in what timelines*
	● None identified
	*Program feature 3 - defining success and measuring it*
	● None identified
Organization	*Program feature 1 - organizing a rapid-response program*
	● Organizations may still lack the skills, structures, processes and a culture to promote and use research findings in decision-making
	*Program feature 2 - deciding what can be done in what timelines*
	● None identified
	*Program feature 3 - defining success and measuring it*
	● None identified
System	*Program feature 1 - organizing a rapid-response program*
	● Decision-makers may be reluctant to rely on a rapid-response program established in another jurisdiction
	● Decision-makers may be reluctant to make requests to an external rapid-response program for politically sensitive issues or to publicly disclose that they made a request
	● Decision-makers may face difficulties in developing a shared vision for a rapid-response program given their constraints and competing priorities
	*Program feature 2 - deciding what can be done in what timelines*
	● Decision-makers may not be inclined to make requests to an external rapid-response program for very short timeframes (e.g. 3 days) given that this may already be done internally on a routine basis
	*Program feature 3 - defining success and measuring it*
	● Decision-makers may be reluctant to fully disclose the impact of the rapid-response program, especially on politically sensitive issues

1. system leaders increasingly working collaboratively to advance the timely translation of research evidence to improve the financing, sustainability and governance of the healthcare system (e.g. Evidence-Informed Healthcare Renewal Roundtable) (program feature 1);
2. the many lessons that have been learned from existing rapid-response programs at the local, national and international levels to decide what can be done in what timeframes (program feature 2); and
3. approaches to evaluation used by other programs that can be built upon to contribute to a broader evidence base about whether and how rapid-response programs work (program feature 3).

### Summary of the dialogue

In addition to the facilitator (JNL) and two members of the McMaster Health Forum team (MGW and FPG), the dialogue brought together a diverse group of 11 stakeholders (nine policymakers/managers and two individuals from organizations that provide support to government decision-makers) from across Canada. Of these, five joined the dialogue in-person and the remaining six joined through video teleconference. The number of dialogue participants was purposefully lower than our typical range of 18 to 22 given our mix of in-person and video participation, and the dialogue was purposefully shorter than our usual full-day dialogue given our smaller number of participants and the challenge of sustaining attention among video participants.

Dialogue participants generally agreed that health system decision-makers faced the three general challenges outlined earlier for finding and using research evidence. During the deliberation about these challenges, participants emphasized five specific challenges. First, the difficulty in accessing the best available research evidence in a timely fashion was highlighted by many. It was specifically noted that the definition of ‘timely’ often differs significantly between researchers and decision-makers, which poses challenges for accessing research evidence through most researchers. Second, contextualizing the research evidence was noted as being challenging but critical for helping to identify what the research evidence means for a particular jurisdiction at a given time. Third, accessing expertise was raised as another challenge, particularly when there is only a limited body of research evidence available from which to draw from. Fourth, participants emphasized the lack of capacity to find and use research evidence (particularly in smaller provinces which may have few internal resources as compared to larger provinces), both in terms of human resources and organizational structures for ‘policy shops’ within ministries of health. Lastly, ensuring the confidentiality of politically sensitive requests (e.g. those linked to negotiations with professional organizations) was seen as a challenge that would need to be addressed (particularly if the products of a rapid-response program are routinely made publicly available).

Dialogue participants agreed with the core components of the three program features and offered several suggestions and/or challenges related to them. First, while agreeing with many of the proposed organizational features in program feature 1, most supported the suggestion from one participant that the initial focus should be on the ‘organic’ development of a pan-Canadian network, with the McMaster Health Forum as a national coordinating hub. With respect to what can be done in what timelines (program feature 2), some questioned whether the three-business-day product would be requested often (given that many ministries of health would do this type of work on their own), whether jurisdictional scans about ‘who’s doing what’ could be offered as a fourth type of product, the feasibility of consistently preparing these products within the proposed timelines and if the products can be translated to make them available in both of the country’s official languages. Lastly, participants noted challenges in the fourth area of program feature 3 (measuring whether and how the product was used). Specifically, several dialogue participants argued that it is difficult to assess the extent to which a product informed or influenced a policy decision. In particular, participants emphasized that the timeline for conducting the evaluation is unlikely to align with the timelines for policy development and decision-making processes (i.e. a decision, or the full sequence of decisions, is unlikely to be made soon after a product is disseminated), thereby limiting the ability to make definitive statements about whether and how a product was used. In addition to the proposed measurement approaches, participants suggested that the possibility of capturing additional data from requestors (before responding to the request) and from others who could benefit from but did not make the request (e.g. download statistics) would be worthwhile to explore.

The main challenges identified for moving forward were securing stable, long-term funding and finding a way to effectively and equitably manage the expected demand. Dialogue participants indicated support for engaging in a process to ‘organically’ build a pan-Canadian network, which they saw as needing to support efforts to 1) develop a common vision for the network, 2) explore how to link with organizations that could contribute to establishing and maintaining the rapid-response network, 3) map potential key individuals and organizations that could be involved within their respective jurisdictions that could contribute to the rapid-response network and identify ways to bring them together, 4) find ‘kindred spirits’ in other provinces and territories (both individuals and organizations) that could join a pan-Canadian rapid-response network and 5) encourage people to direct their health-system-related questions to the newly established rapid-response network.

## Discussion

### Principal findings

We found very few systematic reviews related to the three broad features (organizing a rapid-response program, deciding what can be done in what timelines and defining success and measuring it) of a rapid-response program for health system decision-makers. However, we used an existing analytical framework for how knowledge-brokering organizations can organize themselves [10] to derive possible organizational features (broadly related to governance, management and staffing, program resources and collaborations). We also documented features of other rapid-response programs that are focused on addressing questions related to health systems. In using the issue brief as a starting point for a half-day stakeholder dialogue, the 11 participants from across Canada largely agreed with the content presented in the brief but noted two key challenges to consider: securing stable, long-term funding and finding a way to effectively and equitably manage expected demand. In addition, the main recommendations and suggestions for the next steps from dialogue participants included 1) taking an ‘organic’ approach to developing a pan-Canadian network (with the McMaster Health Forum as the central hub), 2) including jurisdictional scans as a type of product to deliver through the program (rather than only syntheses of research evidence) and 3) refining the evaluation approach to include additional baseline information (e.g. their baseline intention to use research evidence).

### Strengths and limitations

The principal strength of our process is that we paired an issue brief outlining research evidence, analytical frameworks and characteristics of existing rapid-response programs focused on health systems with a robust deliberative process that gave voice to the tacit knowledge and real-world views and experiences of those involved in and/or affected by the issue. The main limitation is that we engaged a relatively smaller number of dialogue participants (*n* = 11) than the 18 to 22 participants that we have previously engaged in other stakeholder dialogues and, hence, may have missed divergent views held by some health system decision-makers across the country.

## Conclusions

Dialogue participants have clearly signalled that there is an appetite for a rapid-response program for health system decision-makers in Canada that addresses problems, options and/or implementation considerations related to a specific health system challenge. In light of the feedback about the need to ‘organically’ build such a program, we are currently engaging in efforts to build partnerships and secure funding to support the creation of a pan-Canadian network for conducting rapid syntheses for health system decision-makers in Canada. This includes the key next steps noted in the summary of the dialogue earlier, namely, developing a common vision and exploring links with other organizations (including funding organizations), networks and ‘kindred spirits’ that could join and contribute to sustaining a pan-Canadian rapid-response network. We believe that building such a network will make a significant contribution to addressing a gap in knowledge translation efforts for health system decision-makers in Canada.

## Additional files

Additional file 1:
**Analysis of organizational features of rapid-response programs targeted to health system decision-makers (table from Wilson et al. 2014) [** 12 **]** **.** The table is a reproduction from Wilson et al. 2014 [12] and provides an analysis of the features of the nine rapid-response programs targeted to health system decision-makers that we identified in the development of the issue brief [1-5,24-28]. Additional file 2:
**Summary of activities of rapid-response programs targeted to health system decision-makers (table from Wilson et al. 2014) [** 12 **]** **.** The table is a reproduction from Wilson et al. 2014 [12] and summarizes the activities of the nine rapid-response programs targeted to health system decision-makers that we identified in the development of the issue brief [1-4,24-30].
