# Supplementary material for: Developing a rapid-response program for health system decision-makers in Canada: findings from an issue brief and stakeholder dialogue
Source: Syst Rev. 2015 Mar 11;4:25. doi: 10.1186/s13643-015-0009-3 (PMC4373100; doi:10.1186/s13643-015-0009-3)
Supplement: Additional file 1: — Analysis of organizational features of rapid-response programs targeted to health system decision-makers (table from Wilson et al. 2014) [ 12 ] . The table is a reproduction from Wilson et al. 2014 [12] and provides an analysis of the features of the nine rapid-response programs targeted to health system decision-makers that we identified in the development of the issue brief [1-5,24-28]. [file 13643_2015_9_MOESM1_ESM.docx]

| Rapid-response program | Features (jurisdiction, target audience and topic focus | Governance | Management and staffing | Program resources | Collaboration |
| --- | --- | --- | --- | --- | --- |
| Canadian Agency for Drugs and Technologies in Health (CADTH) [1] | Jurisdiction   - Canada   Target audience   - Policymakers from federal, provincial and territorial health ministries; health authorities; hospitals; and national and regional healthcare programs   Topic focus   - Drugs and other health technologies | - Administered by CADTH, which is governed by a board of directors that includes provincial and territorial policymakers and managers of health authorities - Provides a clear set of rules and processes for how to make a request, what can be expected, and how the products are developed | - Program has a dedicated manager and is supported by liaison offers for federal, provincial and territorial governments | - Funded internally by CADTH, which is in turn funded by Canadian federal, provincial and territorial governments | - Solicits feedback on projects and drafts reports from a variety of stakeholders |
| Institut national d’excellence en santé et en services sociaux (INESSS) [2] | Jurisdiction   - Québec (Canada)   Target audience   - Policymakers and provider associations   Topic focus   - Drugs and other health technologies; health programs and services; and social programs and services | - Administered by INESSS, which is governed by a board of directors that includes health system managers and researchers - Provides a clear description of what can be expected and how the products are developed (but no description of the rules and processes for making a request) | - The unit has dedicated staff led by the director of the branch and overseen by INESSS vice-president of scientific production | - Funded from within INESSS, which is funded by the Québec government - No information publicly available about the processes to prioritize requests | - External experts are contacted to review scientific aspects of the product to ensure scientific validity of the document produced |
| Ontario HIV Treatment Network (OHTN)^a^ [3] | Jurisdiction   - Ontario (Canada)   Target audience   - Community-based organizations providing services to people with HIV in Ontario   Topic focus   - HIV prevention, support and treatment programs and services | - Administered as a program within the OHTN, which prioritizes the engagement of a range of stakeholders in all of its activities - Uses specified procedures to produce, package and share the rapid syntheses it produces | - Program is overseen by the organizational manager responsible for all synthesis activities - No dedicated staff, but syntheses are conducted by a range of staff with research expertise | - No dedicated funding for the program, but staff resources are drawn from existing programs within the organization - Uses informal processes to prioritize requests | - External experts are often contacted to help identify relevant literature and/or review scientific aspects of the synthesis |
| Ottawa Hospital Research Institute/Champlain Local Health Integration Network (LHIN) [4] | Jurisdiction   - Ontario (Canada)   Target audience   - Managers and stakeholders of the Champlain Local Health Integrated Network   Topic focus   - Disease-management-related programs and services | - Developed and governed through a defined partnership between researchers and a LHIN in Ontario - Uses a defined eight-step process to produce evidence summaries [5] | - Overseen by two researchers and a manager in a LHIN - Program has a dedicated research coordinator to lead the production of evidence summaries (among other responsibilities) | - Funded by a Knowledge to Action grant from the Canadian Institutes of Health Research | - Collaboration between applied health researchers from the Ottawa Hospital Research Institute and the University of Ottawa with the Champlain LHIN |
| Planning Unit of the Ontario Ministry of Health and Long-Term Care (MOHLTC)^a^ | Jurisdiction   - Ontario (Canada)   Target audience   - Policymakers in the divisions, branches and units of the Ontario MOHLTC   Topic focus   - Health system arrangements, programs and services | - Unit is administered by the Planning, Research and Analysis Branch of the MOHLTC - Uses specified procedures to produce, package and share literature reviews produced | - Program has several dedicated staff, is led by a unit manager and is overseen by the director of the branch | - Funded from within the MOHTLC - Uses a process to ensure that the most urgent requests are met | - External experts are engaged where necessary and possible |
| Evidence Check [24,25] | Jurisdiction   - Australia   Target audience   - Health policy and health services agencies   Topic focus   - Health system arrangements, programs and services | - Administered by the Sax Institute, which promotes the use of research evidence in health policy - Provides a clear process for producing a review, including a commissioning process and knowledge-brokering session to clarify the issues/questions to be addressed, and identifying researchers to complete the review | - Knowledge brokers work with policymakers to clarify and refine their policy issues into researchable questions | - Core funding is provided by the New South Wales Ministry of Health with additional funds from other governmental, non-government, philanthropic and competitive research funding agencies | - Reviews are completed by drawing on the Institute’s network of member organizations and researchers and through regular calls for expressions of interest from researchers interested in conducting rapid literature reviews |
| Health Evidence Network (HEN) [26] | Jurisdiction   - Europe   Target audience   - Public health and health system policymakers in the WHO European Region   Topic focus   - Public health policies, and health system policies, programs and services | - Network steering committee advises HEN on its aims, objectives, strategies and approaches | - No information available | - Funding and support provided by the European Commission via the Directorate-General for Health and Consumer Protection, and the Government of France | - Where a detailed synthesis report or a joint policy brief is prioritized, a team of specialists is mobilized to support the development process |
| International Healthcare Comparisons [27] | Jurisdiction   - England   Target audience   - Policymakers in the Department of Health   Topic focus   - Jurisdictional reviews of health system arrangements, programs and services | - Themes focused on in the program are selected in close consultation with the Department of Health in England | - The program is coordinated by a research team based at RAND Europe and the London School of Hygiene & Tropical Medicine | - Funded by the Department of Health in England | - The core team works with experts from a range of countries from the International Healthcare Comparisons Network |
| Supporting the Use of Research Evidence (SURE) Project [28] | Jurisdiction   - Uganda and Burkina Faso (with pilot testing in Cameroon and Zambia)   Target audience   - Policymakers   Topic focus   - Health system arrangements, programs and services | - Program administered by the SURE collaboration, which is a mechanism to support evidence-informed policymaking in Africa | - Program is led by a program officer with SURE who is based out of Makerere University in Uganda | - SURE is funded by the European Commission’s 7th Framework Programme | - SURE project involves teams of researchers and policymakers in seven African countries and is supported by research teams in three European countries and Canada |

^a^Note that the information related to these programs was partially derived based on first-hand experience either from the authors (in the case of the MOHLTC and OHTN programs) or the steering committee (in the case of the MOHTLC program).
