# Supplementary material for: Developing a rapid-response program for health system decision-makers in Canada: findings from an issue brief and stakeholder dialogue
Source: Syst Rev. 2015 Mar 11;4:25. doi: 10.1186/s13643-015-0009-3 (PMC4373100; doi:10.1186/s13643-015-0009-3)
Supplement: Additional file 2: — Summary of activities of rapid-response programs targeted to health system decision-makers (table from Wilson et al. 2014) [ 12 ] . The table is a reproduction from Wilson et al. 2014 [12] and summarizes the activities of the nine rapid-response programs targeted to health system decision-makers that we identified in the development of the issue brief [1-4,24-30]. [file 13643_2015_9_MOESM2_ESM.doc]

| Rapid-response program^a^ | Type of evidence summarized | Products provided (and the timelines in which they are produced) | Key features of products | Volume of production^b^ |
| --- | --- | --- | --- | --- |
| Canadian Agency for Drugs and Technologies in Health [1,29] | - Effectiveness and cost-effectiveness studies evaluating existing healthcare technologies, such as drugs, devices, surgical and nonsurgical procedures, and diagnostic and screening tests. | - Reference list (5 to 10 business days) - Summary of abstracts (15 business days) - Summary with critical appraisal (30 business days) - Peer-reviewed summary with critical appraisal (4 months) - Systematic review and meta-analysis (4 to 5 months) - Rapid health technology assessment (5 to 6 months) - Environmental scan (5 to 40 business days) - Drug review and formulary recommendation (5 to 6 months) - Health technology assessment (case-by-case basis) - Optimal-use project (case-by-case basis) | - Products may be tailored to meet the timeline and needs of the requestor - Developed using systematic and transparent methods for literature searching, study selection, data abstract, synthesis and critical appraisal - Summaries use a structured approach that outline the context of the issue, research questions, key findings, methods used, summary of the evidence and summary of the critical appraisal (when conducted) | - 2,093 products |
| Institut national d’excellence en santé et en services sociaux (INESSS) [2] | - Evidence about effectiveness, safety and other elements requested - In-depth analysis of contextual evidence (e.g. organizational, economic, ethical, legal and social challenges) is not provided | - Reference lists (2 to 3 weeks) - Syntheses of abstracts (3 to 6 weeks) - Briefing notes (3 to 6 months) | - Reference lists (approximately four pages) include summaries of relevant studies with links to relevant documents - A synthesis of abstracts (approximately ten pages) includes a description of the interventions, their effectiveness, utility, safety (and other elements of interest based on a specific request) - A briefing note (approximately 30 pages) includes all of the above but provides a more detailed analysis of these areas based on the available evidence | - Not publicly available |
| Ontario HIV Treatment Network [3] | - Focused on identifying systematic reviews and primary studies when no reviews are available - No restrictions on type of evidence sought (i.e. effectiveness versus non-effectiveness) | - Annotated bibliography (3 to 5 business days) - Rapid reviews (1 month) | - Three- to five-page summaries that follow a standardized reporting approach consisting of key messages, description of the issue, findings, factors that might affect local applicability and methods | - 77 rapid reviews |
| Ottawa Hospital Research Institute [4] | - Primarily effectiveness and sometimes quasi-experimental studies, observational studies and economic analyses - Focused on identifying systematic reviews (single studies included when certain quality thresholds are met, e.g. prospective data collection and rigorous quantitative analysis) | - Evidence summaries (no timeline specified) | - 10- to 15-page summaries that provide key messages, the target audience, relevant disclosures, background, summary of included studies, bottom line statements, a reference list and methods used - Each review and study included in the evidence summaries is assigned a level of evidence, and the quality of systematic reviews is appraised using AMSTAR. | - 16 evidence summaries |
| Planning Unit of the Ontario Ministry of Health and Long-Term Care (MOHLTC) | - Focused on identifying systematic reviews and primary studies (both published and grey literature) when no reviews are available   - No restrictions on type of evidence sought (i.e. effectiveness versus non-effectiveness). - Websites or reports (for jurisdictional scans) | - Literature reviews (3 weeks) - Jurisdictional scans (3 weeks) | - Two to three pages of main messages followed by the limitations of the literature reviewed, time available to produce the review and a footnoted section with detailed findings - Each review is separately checked by another staff member for accuracy, clarity and completeness | - 500 literature reviews and jurisdictional scans (approximately) |
| Evidence Check [24,25] | - Not explicitly stated but the topics addressed suggest no restrictions on type of evidence sought - Evidence is identified through targeted searches for systematic reviews, primary literature, grey literature and jurisdictional scanning | - Evidence Check summaries (no timeline provided on website or the program brochure) | - Reports are approximately 25 to 30 pages and include a two- to five-page executive summary followed by a detailed report that includes an introduction, methods and detailed findings (typically with summary tables) | - 53 Evidence Check reviews |
| International Healthcare Comparisons [27] | - Scope of the program includes responding to specific requests for information on international experience in areas ranging from health sector capacity planning to activity-based financing of hospitals | - Jurisdictional scans (no timeline provided on website) | - Reports are approximately 60 pages in length and include a two-page summary followed by a detailed assessment with an overview of the findings, summary tables and country profiles | - 17 jurisdictional scans |
| Health Evidence Network [26] | - No restrictions on type of evidence sought (i.e. effectiveness versus non-effectiveness) - Evidence is identified through a range of sources: websites, databases, technical and policy documents, national and international organizations and institutions | - Short answer by e-mail (no timeline provided); - One-page evidence summary (no timeline provided - Evidence report - a synthesis of best available evidence in response to a question from a policymaker (no timeline provided) - Joint policy brief - a synthesis of the evidence on a health system problem, policy options for addressing the problem and key implementation considerations (no timeline provided) | - Evidence reports provide a summary that includes an assessment of the issue, findings and policy considerations and a more detailed report that also includes the methods used - Structure of the joint policy briefs vary according to the topic addressed but generally consist of a page of key messages followed by a more detailed assessment of the findings - All reports undergo rigorous external review, as well as internal review - Reports are provided in two or more languages | - 39 evidence reports - 30 policy briefs and summaries |
| SURE Project [28,30] | - Not explicitly stated (although the summary template is focused on approaches to summarizing effectiveness research) | - Rapid synthesis (no specific outline of possible timelines is provided on website, but it does indicate that responses can be provided within 24 to 48 h) | - Uses a standardized structured summary template that provides the key messages, background, details about what was found (including summary tables with assessments of the strength of the evidence) and an assessment of the relevance of the research to the question asked | - 74 (but only 32 in the public domain) |

^a^See Additional file 1 for an overview of the organizational features of each program; ^b^assessed as of 3 February 2014.
